# Respiratory system parameters in children with low severity cystic fibrosis: is there early involvement in relation to healthy peers?

**DOI:** 10.1590/1984-0462/2024/42/2023030

**Published:** 2023-12-11

**Authors:** Gabriela Castilhos Ducati, Juliana Cardoso, Elaine Paulin Ferrazeane, Camila Isabel Santos Schivinski

**Affiliations:** a Universidade do Estado de Santa Catarina, Florianópolis, SC, Brasil.

**Keywords:** Clinical markers, Ventilator-induced lung injury, Spirometry, Oscillometry, Marcadores clínicos, Lesão pulmonar induzida por ventilação mecânica, Espirometria, Oscilometria

## Abstract

**Objective:**

To compare and analyze pulmonary function and respiratory mechanics parameters between healthy children and children with cystic fibrosis.

**Methods:**

This cross-sectional analytical study included healthy children (HSG) and children with cystic fibrosis (CFG), aged 6–13 years, from teaching institutions and a reference center for cystic fibrosis in Florianópolis/SC, Brazil. The patients were paired by age and sex. Initially, an anthropometric evaluation was undertaken to pair the sample characteristics in both groups; the medical records of CFG were consulted for bacterial colonization, genotype, and disease severity (Schwachman-Doershuk Score — SDS) data. Spirometry and impulse oscillometry were used to assess pulmonary function.

**Results:**

In total, 110 children were included, 55 in each group. In the CFG group, 58.2% were classified as excellent by SDS, 49.1% showed the ΔF508 heterozygotic genotype, and 67.3% were colonized by some pathogens. Statistical analysis revealed significant differences between both groups (p<0.05) in most pulmonary function parameters and respiratory mechanics.

**Conclusions:**

Children with cystic fibrosis showed obstructive ventilatory disorders and compromised peripheral airways compared with healthy children. These findings reinforce the early changes in pulmonary function and mechanics associated with this disease.

## RESUMO

**Objetivo:**

Comparar e analisar parâmetros de função pulmonar e de mecânica respiratória entre escolares saudáveis e com fibrose cística (FC).

**Métodos:**

Estudo transversal que incluiu escolares saudáveis (GES) e com FC (GFC), com idades entre seis e 13 anos, provenientes de instituições de ensino e de um centro de referência da FC em Florianópolis/SC, Brasil, pareados por idade e sexo, respectivamente. Inicialmente, conduziu-se avaliação antropométrica para pareamento e caracterização de ambos os grupos e, no GFC, consultou-se prontuário médico para registro dos dados de colonização bacteriana, genótipo e gravidade da doença (Escore de Schwachman-Doershuk — ESD). Para a avaliação da função pulmonar, realizou-se espirometria e a avaliação da mecânica respiratória foi conduzida por meio do sistema de oscilometria de impulso.

**Resultados:**

Participaram 110 escolares, 55 em cada grupo. No GFC, 58,2% foram classificados pelo ESD como excelentes, 49,1% apresentaram genótipo ∆F508 heterozigoto e 67,3% eram colonizados por alguma patógeno. Houve diferença significativa (p<0,05) na maioria dos parâmetros de função pulmonar e de mecânica respiratória entre os grupos.

**Conclusões:**

Escolares com FC apresentaram distúrbio ventilatório obstrutivo e com comprometimento de vias aéreas periféricas, em comparação aos escolares hígidos. Esse evento reforça o início precoce da alteração de função pulmonar e de mecânica respiratória nessa enfermidade, evidenciados pelos achados desta investigação.

## INTRODUCTION

Cystic fibrosis (CF) is a multisystemic disease that affects the pancreatic, hepatic, gastrointestinal, and reproductive systems,^1^ with pulmonary impairment being the leading cause of death. Sputum accumulation, recurrent infections, and chronic inflammation lead to epithelial tissue damage which results in airway remodeling and progressively reduced lung function.^1^ Therefore, disease progression, pulmonary exacerbations, and respiratory insufficiency may require lung transplantation.^2^

For this reason, a systematic assessment of the respiratory system is a part of disease management. Specific examinations using a spirometer and the impulse oscillometry system (IOS) are routinely used to determine the severity of pulmonary impairment, to evaluate the response to therapies, and to monitor disease progression.^3^ Spirometry is indicated for children with CF older than five years and is performed at every clinical appointment or at least twice a year.^3^ Forced expiratory volume in one second (FEV_1_) is the most important parameter because it predicts mortality and lung transplantation.^4,5^ Nowadays, the importance of a forced expiratory flow between 25 and 75% of vital capacity (FEF_25–75%_) is often discussed because it can recognize obstruction in medium and small airways.^3,6,7^

As a supplement to spirometry in CF, IOS analyzes thoracic and pulmonary mechanical properties,^8^ and detects patients with obstructive and restrictive disease. IOS allows passive and straightforward evaluation because it does not involve forced expiratory maneuvers; therefore, it is safe for individuals with CF, as disease progression may lead to airway instability and the technique uses only tidal volume breaths.^9^ IOS measures resistance at 20 Hz (R20), which reflects central airway resistance, and resistance at 5 Hz (R5), which estimates the total airway resistance.^10^ Furthermore, reactance (X) corresponds to the mechanical properties of the distal airways, including alveoli and bronchioles,^10^ whereas pulmonary impedance (Z) is related to the total mechanical load of the entire system.^10^ Therefore, the use of IOS in CF aims to detect early pathologic small airway impairment, similar to the effects of control therapy and response to bronchodilators; thus, IOS is a good tool to control the health status of this disease.^9^

Considering that clinical status worsening is related to impairment of daily living activities, poor quality of life, higher risk of bronchiectasis, and recurrent hospitalization in patients with CF,^2^ comparing data from the respiratory system of healthy, school-age children and those with CF could allow us to understand disease evolution and facilitate early monitoring.^1^ Therefore, the objective of this study was to compare and analyze respiratory system parameters between healthy children and those with CF.

## METHOD

This quantitative cross-sectional study included healthy children (HSG) and a group of children with cystic fibrosis (CFG) aged six to 13 years. The informed consent forms were properly signed by their parents or guardians and approved by the Research Ethics Committee of the State University of Santa Catarina — UDESC (Certificate of Presentation for Ethical Appreciation — CAAE:38770314.1.0000.0118 for healthy children and 36493314.8.000.5361 for the children with CF).

In the CFG, the children were recruited from a referral center for CF in the Joana de Gusmão Children’s Hospital in Florianópolis/SC, Brazil. Diagnosis was confirmed following the Brazilian guidelines for the diagnosis and treatment of CF.^3^ All participants were clinically stable at the time of data collection, according to a Cystic Fibrosis Clinical Score (CFCS)^11^ of less than 25 points, and Cystic Fibrosis Foundation Score (CFFS)^12^ of less than four points, for pulmonary exacerbation.

In both groups, children with diagnosis of cardiorespiratory, musculoskeletal, rheumatic, and neurological disease, as well as those with hearing impairment, visual deficits, assessed through health history questionnaire applied with their guardians, or upper airway symptoms (coryza, sneezing, obstruction of nose, and epistaxis), and those who were unable to perform any of the study procedures were excluded. In the HSG, the children were recruited from teaching institutions and their respiratory health was assessed with the International Study and Allergies in Childhood (ISAAC)^13^ for asthma and allergic rhinitis.

Initially, the sample was characterized by anthropometric evaluation, including body mass measurement in kilograms (Ultra Slim W903-Wiso^®^) and height in meters (Sanny^®^). The children were in a standing position, wearing light clothes and no shoes for this evaluation. To calculate body mass index (BMI-kg/m^2^) a children’s calculator available at the Ministry of Health/Brazil Telehealth Program website^14^ was used. In the CFG, pathogen colonization data, genotype, disease severity, and the Schwachman-Doershuk score (SDS) were obtained from the medical records. The SDS classifies disease severity as severe, poor, average, good, and excellent when the scores are <40, 40–55, 56–70, 85–71, and 86–100, respectively.^15^

To assess pulmonary function, spirometry was performed using a portable spirometer (EasyOne^®^, Fleximed, USA) following the American Thoracic Society/ATS guidelines.^16^ To execute the test, each child remained seated while wearing a nasal clip and performed a forced expiratory maneuver three times. The parameters of forced vital capacity (FVC), forced expiratory flow in one second (FEV_1_), forced expiratory flow at 25–75% (FEF_25-75%_), and peak expiratory flow (PEF) were considered as absolute and predicted percentage (%pred) values, according to Polgar and Weng^17^ and Knudson et al.^18^

Mechanical respiratory assessment was performed using the IOS (pneumatograph Master Screen IOS, Erich Jaeger, Germany^®^), the calibration was completed daily and the device was located in a controlled environment (temperature and relative humidity), monitored through the digital Thermo hygrometer Incoterm 7663^®^, ensuring temperatures between 17 and 40°C.

The exam was performed following the ATS guidelines.^19^ The children remained in a sitting position and were instructed to couple their mouth to the mouthpiece and to perform spontaneous breaths in tidal volume, stable and smooth. Oscillometric measures of at least 20 seconds were considered. The test was considered valid if the trace was linear, ascendant, and within the system’s range of normality and when the child performed the test without interference such as coughing, crying, or swallowing during measurements. All participants performed three consecutive measurements, with a standard interval of 30 s between each measurement.

The registered absolute and predicted percentage values of respiratory impedance at 5 Hz (Z5), total airway resistance (R5), central airway resistance (R20), and reactance at 5 Hz (X5) parameters were considered as absolute and predicted percentage (%pred) values calculated according to the Brazilian equation.^20^

Data were analyzed using the Statistical Package for the Social Sciences (SPSS^®^) version 20.0. Data distribution was verified using the Shapiro-Wilk test, applying descriptive statistics and frequencies. In addition, the Mann-Whitney U test was used to compare the respiratory system parameters between groups. The significance level was set at 5% for all the tests. The sample size was calculated *a priori* using G*Power 3.1 software, with the test power set at 95%, effect size of 0.80 — adopted based on the premise that with this value we can guarantee enough power to detect any effect that may have existed^21^ — and the significance level set at 5%, estimating 55 children in each group.

## RESULTS

In total, 110 children participated in this study (55 in each group). In CFG, 58.2% were classified as excellent by SDS, 49.1% showed the ΔF508 heterozygotic genotype, and 67.3% were colonized by some pathogens. The other sample characterization data are shown in Table 1.

**Table 1.** Sample characterization regarding sex, age, anthropometry, pulmonary function, respiratory mechanic parameters, and comparison results between the healthy children and children with cystic fibrosis

Variables	HSG n=55	CFG n=55	p-value
	Mean±SD (CI)	Mean±SD (CI)	
Age (years)	9.45±2.18 (8.86–10.04)	9.52±2.26 (8.91–10.14)	0.860
Female sex (%)	45.5	45.5	-
Body mass (kg)	31.90±9.16 (29.43–34.38)	30.09±9.20 (27.60–32.58)	0.180
Height (m)	1.40±0.17 (1.36–1.45)	1.35±0.14 (1.32–1.39)	0.260
BMI (kg/m²)	16.07±2.25 (15.46–16.68)	15.91±2.21 (15.31–16.51)	0.758
Z5 (kPa/L/s)	0.63±0.15 (0.59–0.67)	0.82±0.31 (0.74–0.91)	<0.01
R5 (kPa/L/s)	0.60±0.15 (0.56–0.64)	0.75±0.28 (0.68–0.83)	<0.01
R20 (kPa/L/s)	0.53±0.40 (0.42–0.64)	0.52±0.10 (0.49–0.55)	0.102
X5 (kPa/L/s)	-0.17±0.05 (-0.19–(-0.16))	-0.32±0.17 (-0.37–(-0.27))	<0.01
Z5 (%)	141.78±43.70 (129.96–153.59)	182.05±81.61 (159.98–204.11)	0.010
R5 (%)	97.06±22.64 (90.94–103.18)	121.50±44.58 (109.45–133.56)	<0.01
R20 (%)	90.84±19.81 (85.48–96.20)	100.11±19.57 (94.82–105.40)	0.029
X5 (%)	125.99±38.02 (115.71–136.27)	216.55±124.65 (182.85–250.25)	<0.01
FVC (L/min)	2.28±0.81 (2.06–2.50)	1.83±0.67 (1.65–2.01)	<0.01
FEV_1_ (L/min)	2.71±1.15 (2.40–3.02)	1.42±0.64 (1.24–1.59)	<0.01
FEF_25-75_ (L/min)	2.38±0.81 (2.16–2.60)	1.25±0.79 (1.04–1.47)	<0.01
PEF (L/min)	3.83±1.37 (3.46–4.20)	3.01±1.29 (2.66–3.36)	<0.01
FVC (%)	95.81±18.09 (90.92–100.70)	84.70±23.09 (78.46–90.94)	<0.01
FEV_1_ (%)	91.52±14.03 (87.73–95.32)	71.60±24.84 (64.88–78.31)	<0.01
FEF_25-75_ (%)	89.05±22.09 (83.08–95.03)	52.29±30.06 (44.17–60.42)	<0.01
PEF (%)	87.15±15.71 (82.90–91.40)	64.27±23.69 (57.87–70.68)	<0.01

HSG: healthy schoolchildren; CFG: cystic fibrosis; N: number of participants; SD: standard deviation; CI: confidence interval; Z5: impedance at 5 Hz; kPa/L/s: kilopascal per liter per second; R5: total airway resistance; R20: central airway resistance; X5: reactance at 5 Hz; FVC: forced vital expiratory capacity; FEV_1:_ forced expiratory volume in one second; FEF_25-75_: forced expiratory flow between 25–75% of FEV; PEF: peak expiratory flow; L/min: liter per minute; Kg: kilogram; M: meters; Kg/m²: kilogram per square meter; p-value: statistical significance value.

Regarding pulmonary function, the CFG had FEV_1_, FEF_25–75%_, and PEF% below the predicted value, and there was a significant difference between the groups in all spirometric parameters assessed (p<0.05). Further, most mechanical respiratory parameters, except R20 (Table 1), showed a difference between the CFG and HSG, with higher values for CF. Furthermore, the HSG had spirometric and oscillometric parameters in the predicted range of values, except Z5 and X5 that were above the predicted values.

## DISCUSSION

This study demonstrates the significant differences between the respiratory system parameters of children with CF and their healthy counterparts, a statement that is supported by the literature,^7,10^ reinforcing the importance of early monitoring disease progression using this system. Despite the low disease severity in the study sample, pulmonary function and respiratory mechanics of children with CF were already altered in the school age as compared to healthy children, which is worthy of attention. Furthermore, most children with CF had obstructive ventilatory disorders and increased airway resistance, mainly in the peripheral airways, which corroborates findings previously published in the literature^22^ concerning the early and progressive respiratory system impairment, with an initial impact in small airways.

In this context, FEV_1_ is one of the main predictors of pulmonary function decline,^23^ whereas, in the current investigation, the CFG had values below 80%, despite a low disease severity. Furthermore, according to the literature, adolescents are at a higher risk of decline in pulmonary function than are children or adults.^24^ A longitudinal study of 20,664 patients with CF concluded that the propensity for a decline in pulmonary function increases progressively from six to 15 years of age.^24^ A later study suggested that children with worse pulmonary function in early infancy (from the age of six to eight) tend to have higher declines during childhood and adolescence,^25^ ultimately leading to inadequate ventilation distribution, airway obstruction, pulmonary hyperinflation, and air trapping.^22^

However, a systematic review showed that the predicted decline in FEV_1_ with age is not static or directly proportional, as discussed previously, but dynamic and variable over time.^26^ This statement is justified when disease progression is considered to be associated not only with physiological factors but also with the influence of the social environment (economic position, education, family and cultural beliefs) of each individual.^27^ This scenario reinforces the importance of an early systematic assessment of pulmonary function, considering all spheres in which the individual is active, so that it is possible to act more consistently throughout the disease progression, at different ages, but mainly in younger children.

It is also necessary to consider other spirometric parameters. Studies have shown that parameters such as FEF_25–75%_ and FEV_1_/FVC are sensitive enough to identify obstruction in small airways.^6^ In a study conducted by American researchers, including 93 children with CF at a mean age of four years, FEF_25–75%_ was already reduced when compared with that in a control group.^7^ This corroborates the current findings, which detected a difference in this parameter between the analyzed children. The FEF_25–75%_ measures the mean flow in a determined volume interval, including small- and medium-caliber airways,^28^ which are frequently impaired in CF.^29^ In addition, the HSG did not have respiratory disorders. A study with 1.990 healthy Brazilian children identified that most were within the predicted value in the FEV_1_ and FVC parameters.^30^

As a complementary analysis, IOS is included in routine assessments at big referral centers for CF,^31^ as it only requires passive cooperation and is more acceptable among younger children. It differentiates resistance in central and peripheral components of the airway and detects early pathological changes in small-caliber airways.^9^ Furthermore, it is helpful to assess respiratory mechanics and to monitor acute pulmonary exacerbations and has been used as an alternative to characterize the level of impairment in pulmonary function in the initial disease stage.^31^

Similar to the current research, a study that included 190 individuals aged between six and 14 years compared IOS results between CF and healthy subjects and found significant differences in respiratory mechanics parameters, except for R20.^10^ In contrast, Sakarya et al. found significant differences in all IOS parameters between CF patients and healthy children (R5-10-15-20 Hz), as well as Z5, Fres, and AX, which were all higher in the CFG than in the healthy group.^31^ These alterations are consistent as pulmonary impairment begins in early infancy, and distal airways involvement plays a predominant role in disease progression.^22^

Walter et al. suggested that 50% of lung function decline is explained by pulmonary exacerbations (PEs),^32^ while other studies concluded that this impairment is also associated with nutritional status, airway clearance, and bronchiectasis.^3,4^ Therefore, interventions such as inhalation therapy and the airway clearance physiotherapy (ACP) are important to prevent PEs and to delay the decline in lung function.^3^ The ACP should be performed daily after the diagnosis in all patients with CF, through a variety of airway clearance techniques, including conventional chest physiotherapy (postural drainage, percussion, and vibration), active cycle of breathing techniques, autogenic drainage, high-pressure PEP therapy, and oscillatory devices. However, current literature reports no superiority of any technique over the others and the choice must be individualized.^3,4^

In conclusion, the results obtained from our study demonstrate that children with CF showed obstructive ventilatory disorders and compromised peripheral airways compared with healthy children.
